# Nonsusceptibility of Primate Cells to Taura Syndrome Virus

**DOI:** 10.3201/eid1012.040419

**Published:** 2004-12

**Authors:** Carlos R. Pantoja, Solangel A. Navarro, Jaime Naranjo, Donald V. Lightner, Charles P. Gerba

**Affiliations:** * University of Arizona, Tucson, Arizona, USA

**Keywords:** Taura syndrome virus, cripavirus, dicistroviridae, picornaviridae, bgmk, ma-104, frhk-4, real time rt-pcr, litopenaeus vannamei, research

## Abstract

Taura syndrome virus (TSV), a pathogen of penaeid shrimp and member of the family *Dicistroviridae*, was recently reported to have the ability to infect primate cells. We independently retested this hypothesis. Three lines of primate cells FRhK-4, MA-104, and BGMK, which are highly susceptible to infection by human picornaviruses, were challenged with TSV. Viral replication was assayed by real-time reverse transcription–polymerase chain reaction using cell media samples collected on days 0, 4, and 7 postchallenge. By day 7, genome copy numbers had decreased 25%–99%. No cytopathic effect was observed after 7 days. An in situ hybridization assay, with gene probes specific for detection of TSV, was negative for TSV in challenged cells. The infectivity of residual virus in the cell culture media at day 7 was confirmed by bioassay using TSV-free indicator shrimp (*Litopenaeus vannamei*). TSV did not infect the primate cells tested, and no evidence of zoonotic potential was found.

## Abstract 2

Primate cells commonly used to test for viruses of the *Picornaviridae* family are not susceptible to infection by Taura syndrome virus of penaeid shrimp.

The general assumption is that the viral agents that cause disease in penaeid shrimp do not infect vertebrates. Supporting this assumption is the absence of documented cases of any shrimp virus causing disease in any animal species other than crustaceans. In a recent article, Taura syndrome virus (TSV), exclusively a pathogen of penaeid shrimp, was attributed a zoonotic potential because of its reported ability to infect cultured human and monkey cells (*1*). Aside from the food safety issues raised by this report, we were very interested in confirming those results because of the practical value of this new option for growing TSV in vitro. To date, TSV (or any other of the known viruses of the penaeid shrimp) has not yet been successfully cultured in any invertebrate or vertebrate cell-culture system. Hence, if viable, the use of primate cells for propagation of TSV would prove to be an important advancement in the study of TSV, and perhaps of other crustacean viruses. While the experiment reported in this study did not include all of the cell lines used by Audelo-del-Valle et al. (*1*), namely human rhabdomyosarcoma (RD), human larynx carcinoma (Hep-2), and Buffalo green monkey kidney (BGMK), the three cell lines that we used are also routinely used for virus isolation and diagnosis of diseases caused by human enteroviruses that belong to the family *Picornaviridae* (*2**–**7*). TSV, is a member of the family *Dicistroviridae* (closely related to *Picornaviridae*), genus *Cripavirus* (*8**,**9*). Other than TSV, which only infects penaeid shrimp, members of the genus *Cripavirus* are known to infect only insects (*9*). We report the results obtained after performing an experiment to test the hypothesis proposed by Audelo-del-Valle et al. (*1*).

## Materials and Methods

### Source of TSV

Rather than using TSV-infected shrimp originated from shrimp farms as a source, TSV infection was induced under laboratory conditions by injecting specific pathogen free (SPF) shrimp (*10*) *Litopenaeus vannamei* with purified TSV reference isolate Hawaii-94 (*11*). The use of SPF shrimp ensured that no contamination with other viral pathogens would interfere with the experiment. Hemolymph and hepatopancreas were obtained from moribund shrimp during the acute phase of Taura syndrome and used to prepare the viral inocula.

### Preparation of Inocula

Hemolymph was drawn from a moribund shrimp with acute-phase Taura syndrome and the hepatopancreas was excised by using aseptic technique. The hemolymph was diluted 1:10 with Eagle's balanced salts minimum essential medium (EMEM), without fetal bovine sera (FBS), and filtered through a syringe filter of 0.22-μm pore size. The hepatopancreas was homogenized in 10 mL of EMEM without FBS, centrifuged at 125 x *g* for 2 min to eliminate coarse material and the supernatant filtered with a syringe filter of 0.22-μm pore size. Samples of hemolymph and hepatopancreas from SPF shrimp were processed in identical manner and used as a negative control.

### Cell Culture

The lines and cell cultures used were African green monkey kidney (BGMK), Monkey Rhesus female kidney embryonic (FRhK-4), and Monkey African green kidney (MA-104). Other than the report by Audelo-del-Valle et al. (*1*), TSV culture or CPE has not been reported in any invertebrate or vertebrate cell line. Hence, no positive control for TSV-induced CPE in cell culture was included in this study.

### TSV Injection

Each of four 75-cm^2^ flasks of confluent monolayers of each cell line (BGMK, FRhK-4, and MA-104) was injected with 0.1 mL of either of the four inocula: 1) hemolymph from shrimp with acute-phase Taura syndrome, 2) hepatopancreas from shrimp with acute-phase Taura syndrome, 3) hemolymph from SPF shrimp, or 4) hepatopancreas from SPF shrimp. After injection, the standard volume (15 mL) of fresh EMEM with 2% FBS was added without removing the inoculum. The cells were incubated at 37°C and monitored once a day for 7 days for cytopathic effect (CPE). As an additional negative control, one flask of each of the three cell lines was left untreated but monitored once a day alongside the TSV-injected flasks.

As an additional test to determine if a productive TSV infection occurred, representative samples of cells at day 7 were collected with a sterile pipette and pelleted at 130 x *g*. The pellet of cells was fixed in Davidson's AFA (alcohol, formaldehyde, and acetic acid) fixative and processed by using conventional techniques for paraffin embedding and sectioning. Paraffin sections were subjected to in situ hybridization with a mixture of two gene probes, P15 and Q1, specific for detection of TSV (*12*), according to protocols published elsewhere (*13**,**14*).

### TSV Quantification

#### RNA Extraction

A total of 0.2 mL from each of the original inocula (inocula prepared from the hemolymph and hepatopancreas of infected and noninfected shrimp) and 0.2 mL of cell culture media from each of the three different cell line cultures collected at days 0, 4, and 7 postexposure, were subjected to RNA extractions using a High Pure RNA tissue kit (Roche Molecular Biochemicals, Indianapolis, IN), according to the manufacturer's recommendations. The concentration of extracted RNA was estimated by measuring optical density, OD_260nm_, with an Eppendorf spectrophotometer.

#### Real-Time TSV RT-PCR

The real time TSV RT-PCR assays were performed using an ABI GeneAmp 5700 with TaqMan One-Step RT-PCR master mixture (Applied Biosystems, Foster City, CA). The reaction mixture contained no more than 10 ng of extracted RNA, with each primer at a concentration of 0.3 μmol/L, and the TaqMan probe at a concentration of 0.1 μmol/L in a final volume of 25 μL. The cycling consisted of 30 min at 48°C for reverse transcription and 10 min at 95°C, followed by 40 cycles of 95°C for 15 s, and 60°C for 1 min. The data acquisition and analysis were carried out with GeneAmp 5700 Sequence Detector Software (Applied Biosystems). The real-time RT-PCR primers and TaqMan probe for the detection of TSV had been previously designed from ORF1 region of the TSV genomic sequence (*15*). Serial dilutions from a previously constructed TSV plasmid were used to determine a standard linear relationship for quantification with a correlation of the serial dilutions >0.99.

### Confirmation of TSV Infectivity

#### Bioassay

To confirm the infectivity of the virus, a 6-day bioassay was performed by injecting groups of four to six SPF indicator shrimp (*L. vannamei*) with approximately 200 μL of either of the following: 1) inoculum prepared from hemolymph of infected shrimp; 2) inoculum prepared from hemolymph of noninfected shrimp; 3) cell media collected at day 7 from TSV-challenged cell culture flasks; or 4) culture media collected at day 7 from SPF shrimp tissue–treated cell culture flasks (negative control). The shrimp were monitored once a day for signs of disease. Moribund shrimp were collected when observed, preserved in Davidson's fixative and transferred into 70% ethanol after 24 h (*14**,**16*). All surviving shrimp at termination of the bioassay (day 6) were preserved in the same manner. Shrimp tissue samples were processed according to conventional techniques for paraffin embedding and sectioning (*16*). Paraffin sections were stained with Mayer-Bennett's hematoxylin/eosin-phloxin and subjected to histologic evaluation to determine the presence of TSV diagnostic lesions. Selected specimens were subjected to a confirmatory assay by in situ hybridization (ISH) with a mixture of two gene probes, P15 and Q1, specific for detection of TSV (*12**–**14*).

## Results

### Cytopathic Effect (CPE)

**Figure 1:**
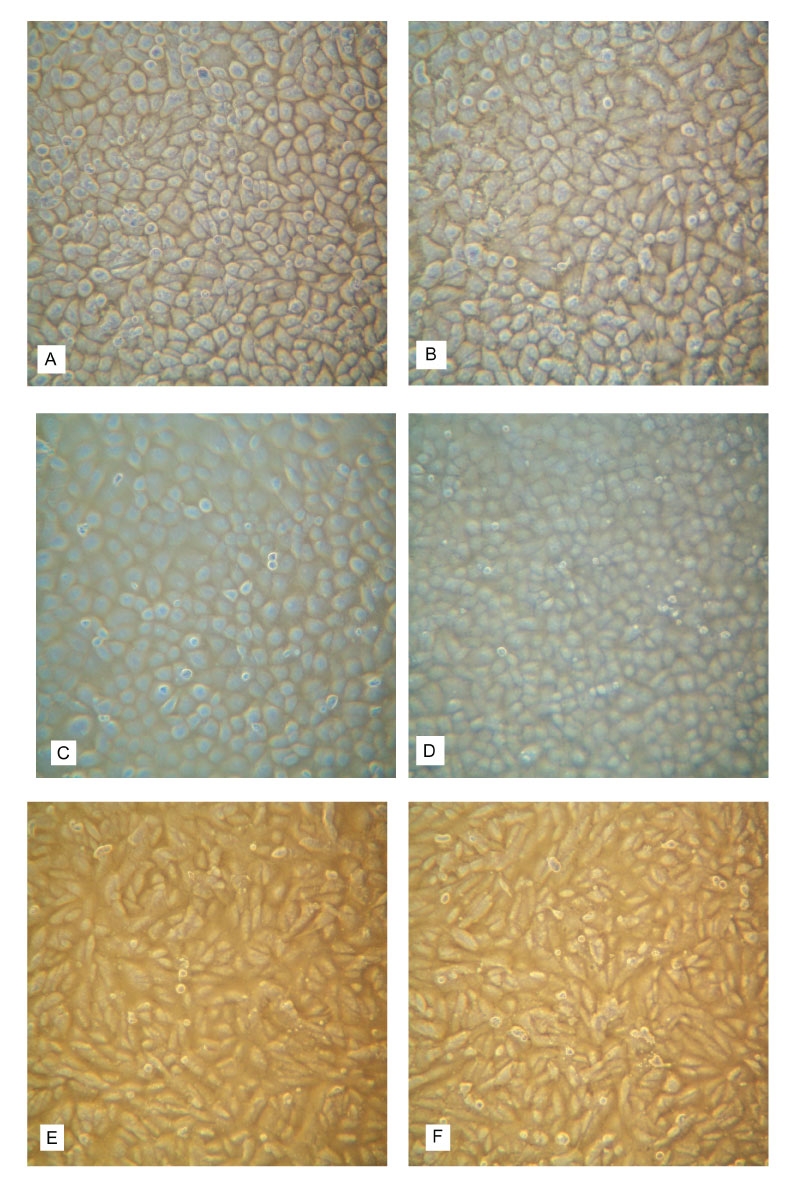
Absence of cytopathic effect after a 7-day exposure of three lines of primate cells (FRhK-4, MA-104, and BGMK) to an inoculum prepared with hemolymph from Taura syndrome virus (TSV)-infected shrimp (*Litopenaeus vannamei*) during the acute phase of the disease compared to a control inoculum containing hemolymph from specific pathogen free (SPF) shrimp. A) FRhK-4 cells exposed to SPF hemolymph; B) FRhK-4 cells exposed to TSV hemolymph; C) MA-104 cells exposed to SPF hemolymph; D) MA-104 cells exposed to TSV hemolymph; E) BGMK cells exposed to SPF hemolymph; F) BGMK cells exposed to TSV hemolymph (no stain; 25x).

No CPE was observed in any of the three cell lines injected with TSV infected hemolymph, TSV infected hepatopancreas, SPF shrimp hemolymph, or SPF shrimp hepatopancreas (Figure 1). The BGMK cell line showed normal fibroblastic structure throughout the 7-day period of exposure to the different treatments. The BGMK monolayer remained confluent with no evidence of cell detachment or lysis. Similarly, the FRhK-4 and the MA-104 cell lines retained their typical epithelial structure for the 7-day period after exposure to TSV, with confluent monolayers and no evidence of cell detachment or lysis.

### Virus Quantification

Approximately 1.3 x 10^5^ to 2.7 x 10^6^ viral copies/μL were detected at day 0 in the tissue cell flasks exposed to TSV-infected hemolymph. In the case of the tissue cell flasks exposed to TSV-infected hepatopancreas, ≈1.2 x 10^4^ viral copies/μL were detected (Table). TSV was not detected by real time RT-PCR in the inoculum prepared from SPF shrimp hemolymph and hepatopancreas, nor in the tissue cell culture flasks exposed to these inocula.

**Table:** Mean TSV quantification in tissue cell culture media^a,b^

Cell type	Source of inoculum	Inoculum type	Mean viral quantification (viral copies/μL) postexposure		
			Day 0	Day 4	Day 7
BGMK	Hemolymph	TSV-infected	2.7 x 10^6^	2.4 x 10^5^	2.5 x 10^4^
		SPF^c^	0	0	0
	Hepatopancreas	TSV-infected	1.2 x 10^4^	5.0 x 10^3^	5.0 x 10^3^
		SPF	0	0	0
FrhK-4	Hemolymph	TSV-infected	1.3 x 10^5^	1.9 x 10^4^	4.7 x 10^3^
		SPF	0	0	0
	Hepatopancreas	TSV-infected	1.3 x 10^4^	8.4 x 10^3^	3.7 x 10^3^
		SPF	0	0	0
MA-104	Hemolymph	TSV-infected	1.5 x 10^5^	3.1 x 10^4^	2.3 x 10^3^
		SPF	0	0	0
	Hepatopancreas	TSV-infected	1.2 x 10^4^	1.3 x 10^4^	9.2 x 10^3^
		SPF	0	0	0

^a^Estimated by real time reverse transcription–polymerase chain reaction at different intervals postexposure. ^b^BGMK, Buffalo green monkey kidney; FrhK-4, monkey *Rhesus* female kidney embryonic; MA-104, monkey African green kidney; TSV; Taura syndrome virus; SPF, specific pathogen free. ^c^Inoculum originated from SPF penaeid shrimp.

**Figure 2:**
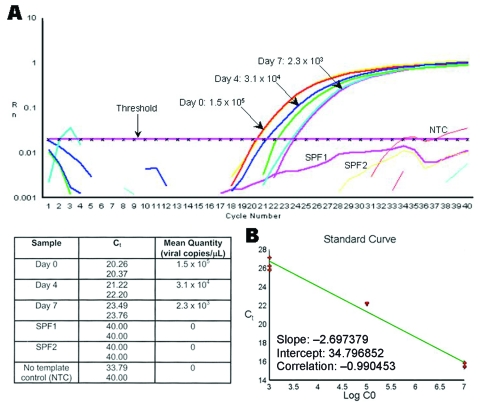
Example of the decrease on Taura syndrome virus (TSV) genome copy number within tissue cell culture flasks exposed to TSV. A) Real time reverse transcription–polymerase chain reaction plots and mean quantity of TSV copies/μL from tissue cell culture media collected at days 0, 4, and 7 postinfection from MA-104 cell flasks injected with TSV-infected shrimp hemolymph. Samples of tissue cell culture media collected from FrhK-4 and BGMK cell culture flasks inoculated with TSV-infected hemolymph or hepatopancreas also decreased by >1 log in concentration of viral copies as a function of time. The value of 33.79 obtained for one of the no template control (NTC) replicates is considered an artifact. B) Standard curve of TSV copy number versus threshold cycle (C_t_) value. Purified TSV plasmid was serially diluted and used as templates in real-time polymerase chain reaction. The resulting C_t_ values are plotted against the logarithm of their respective copy numbers (C0). R_n,_ fluorescence signal; SPF 1 and 2, specific pathogen free.

At day 7, real time RT-PCR showed a decrease of 25% to 99% of the TSV genome copy number in the tissue cell culture flasks exposed to TSV (Figure 2), which suggests that no viral replication had occurred but that some residual virus remained from the inoculum.

### Bioassay

**Figure 3:**
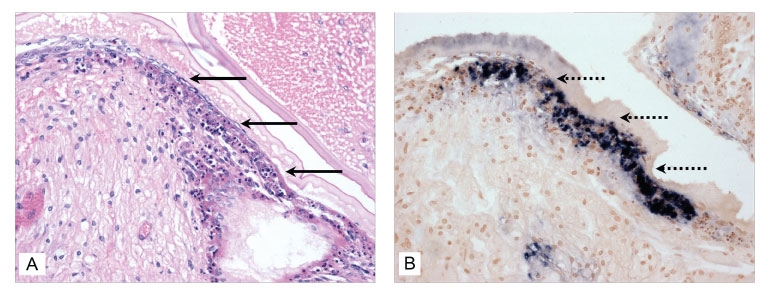
Histologic section through the anterior gastric chamber of a moribund juvenile *Litopenaeus vannamei* that was injected with an inoculum prepared with tissue cell culture media from BGMK cells exposed to Taura syndrome virus (TSV) (day 7 postexposure). A) The arrows point to a portion of cuticular epithelium displaying diagnostic acute-phase TSV lesions (hematoxylin/eosin-phloxin stain; 50x). B) The dashed arrows point to a portion of the stomach epithelium from the same shrimp, where digoxigenin (DIG)-labeled TSV-specific gene probes were reacted by in situ hybridization (ISH), resulting in the deposition of a black precipitate on areas where the probe hybridized with target TSV (Bismarck Brown counterstain; 50x).

Samples of cell-culture media from tissue culture flasks injected with TSV-infected hemolymph were collected at day 7 and used to inject SPF indicator shrimp *L. vannamei* to determine the infectivity of the residual virus. Moribund shrimp from these bioassays were examined by conventional hematoxylin/eosin-phloxin histology and by in situ hybridization with the gene probes specific for detection of TSV. TSV infection was diagnosed in all of the moribund shrimp, which indicates that at 7 days after injection, the tissue culture media contained sufficient residual TSV to produce infections in challenged shrimp (Figure 3). Paraffin sections from known TSV-infected and noninfected shrimp were used as ISH positive and negative controls, respectively (results not shown).

**Figure 4:**
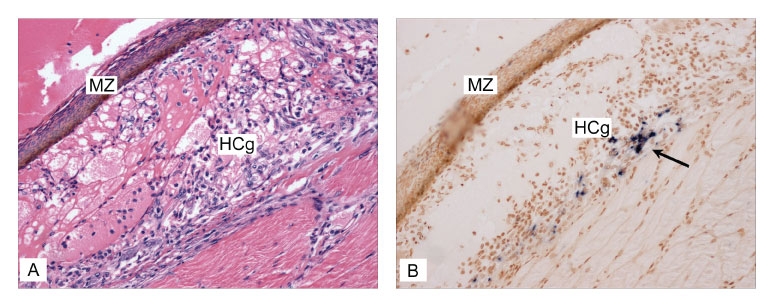
Covert Taura syndrome virus (TSV) infection (transition/chronic phase of TS disease) in indicator specific pathogen free–*Litopenaeus vannamei* shrimp was confirmed by in situ hybridization (ISH) with digoxigenin-labeled gene probes specific for detection of TSV. A) Histologic section through the dorsal cuticular epithelium showing a melanized resolving lesion (MZ) and hemolytic congestion (HCg), indicative of the transition phase of TSV infection (hematoxylin/eosin-phloxin stain; 50x) . B) TSV ISH on the consecutive section to that shown in 5A, where binding of the TSV probes is shown by the black precipitate (arrow) indicating the presence of TSV within the cytoplasm of cells of the cuticular epithelium (Bismarck Brown counterstain; 50x)

During this study, a relationship was observed between the concentration of TSV in the inocula prepared from day 7 tissue culture media (from cells exposed to TSV-infected hemolymph) and the severity of TSV infection in the challenged SPF indicator shrimp. The shrimp that had been injected with tissue cell culture media with the highest TSV concentration (≈2.5 x 10^4^ viral copies/μL) developed an acute (overt) infection within 3 days postchallenge, whereas shrimp injected with tissue cell culture media with the lowest viral concentration (≈2.3 x 10^3^ viral copies/μL) developed only a subacute (covert) infection. Both overt and covert infections were confirmed by histologic analysis and by ISH with gene probes specific for detection of TSV (Figure 4).

### Additional ISH Test

**Figure 5:**
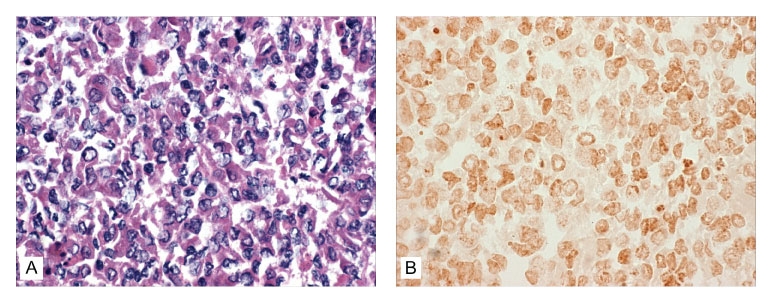
Absence of reaction by in situ hybridization (ISH) to the digoxigenin (DIG)-labeled Taura syndrome virus (TSV) probes within the BGMK cells harvested at day 7 postinjection with TSV. A) No cytopathic effect suggestive of TSV infection was evident by conventional hematoxylin/eosin-phloxin (H&E) histology (H&E stain; 100x). B) Consecutive histologic section to that shown in Figure 4A, but subjected to ISH with DIG-labeled TSV probes specific for TSV. No reaction to TSV is apparent in the challenged cells (Bismarck Brown counterstain; 100x).

As an additional test to further confirm the absence of viral replication, the accumulation within the cells, or both, a sample of cells at day 7 was obtained from the BGMK cell line and subjected to ISH with TSV-specific gene probes. The BGMK cell line was selected for this assay because, among all three lines, it had the highest initial (day 0) concentration of viral particles and a 2 log reduction at day 7, suggesting either degradation or internalization of the virus. The ISH assay gave negative results (Figure 5), which indicates degradation as the more likely explanation for the reduction in virus content of the cell media. As in previous ISH assays, paraffin sections from known TSV-infected and noninfected shrimp were used as ISH-positive and -negative controls, respectively (results not shown).

## Discussion

The real-time RT-PCR results (Table) show that the number of TSV genome copies in the cell culture media did not increase for any of the three cell lines challenged with TSV. While differences were observed in the estimated number of viral copies in each flask at day 7, the number of viral copies present was from one to two logs less than that of the day 0 values, which were determined immediately after cells were injected. The apparent plateau of TSV counts at day 7, regardless of the concentration of viral particles in each flask at day 0, may have been due to a protective effect of the cell-culture media, specifically the fetal bovine serum (FBS). This protective effect of FBS on viruses has been documented by other researchers (*17**–**20*). Studies on viral transport media for the preservation of virus viability have concluded that the best transport media for specimens at risk of being delayed by long transit times and exposed to significant temperature variations en route, are those that contain 2% FBS (i.e., CVM-Copan Diagnostics, Corana, CA, and M4-Multi-Microbe, Micro Test, Inc., Snellville, GA).

To further confirm the absence of viral replication or accumulation within the cells, a sample of cells at day 7 was obtained from the BGMK cell line and subjected to ISH with TSV-specific gene probes. The absence of reaction to the TSV probes and the absence of CPE indicate that viral replication did not occur in the BGMK cells and that if any viral particles were internalized, they were degraded.

The relationship observed between the concentration of TSV in the inocula prepared from day 7 tissue culture media (from cells exposed to TSV-infected hemolymph) and the severity of TSV infection in the challenged SPF indicator shrimp agree with the results obtained during a previous study in the authors' laboratory, in which it was concluded that a minimum concentration of ≈1.0 x 10^4^ viral copies/μL is necessary to induce an acute infection (*21*). In this case, those shrimp that had been injected with tissue cell culture media with the highest TSV concentration (≈2.5 x 10^4^ viral copies/μL) developed an acute (overt) infection within 3 days postchallenge, whereas shrimp injected with tissue cell culture media with the lowest viral concentration (≈2.3 x 10^3^ viral copies/μL) developed only a subacute (covert) infection.

BGMK, FRhK-4, and MA-104 cell lines are often used for isolation and research purposes for enteroviral meningitis (*3*), hepatitis A virus (*7*), polioviruses, coxsackie A, and coxsackie B (*22*) because of their marked susceptibility to infection by these members of the *Picornaviridae*. When exposed to any of these agents, these cell lines develop conspicuous CPE within ≈5 days (*3**,**7**,**22**,**23*). However, no CPE in any of the three cell lines (BGMK, FRhK-4, and MA-104) challenged with TSV was observed in this experiment, even at day 7 postinjection. These results contradict those of Audelo-del-Valle et al. (*1*), who reported the development of CPE within 19–23 hours. The average incubation time required for CPE development (induced by enteroviruses) in human or monkey cells at 37°C is 5 days, although detection time may be reduced to ≈3 days by use of the shell vial method (*23*). Shorter incubation times of <24 hours for CPE development could be more suggestive of a toxicity problem rather than of virus induced CPE.

As mentioned above, SPF shrimp (*L. vannamei*) were used to amplify a reference strain of TSV to prepare the inocula. We used SPF shrimp for three reasons. First, pond-reared or wild shrimp may harbor human or other mammalian picornaviruses. Shrimp and other decapod crustaceans have been shown to internalize and passively carry certain fish viruses (*24**–**26*) and human enteroviruses (*27*, C. Gerba, unpub. data). Hence, wild or pond-reared shrimp may be passive carriers of human or other mammalian pricornaviruses or other viruses which could produce CPE in studies such as that reported by Audelo-del-Valle et al. (*1*). Second, by using a commercially available line of SPF shrimp, the experiment can be standardized; therefore, other researchers can repeat or confirm the present study. Third, the SPF shrimp used are produced in closed biosecure systems with controlled water sources, which preclude chance contamination of the stocks with human or other animal viruses.

BGMK cells were the only cell type in common between our study and that of Audelo-del-Valle et al (*1*), who also used RD and Hep-2 however, BGMK, FRhK-4, and MA-104 cells were selected for use in our study because these cell types have a marked susceptibility to infection by members of *Picornaviridae* (*28*), which makes them as adequate as RD or Hep-2 cells for determining the possible infectivity of TSV to primate cells. We conclude that TSV did not infect the primate cells challenged with TSV in our study.

The lack of CPE in any of the three different cell lines tested, the negative ISH results with TSV specific gene probes assay of TSV challenged BGMK cells, and multilog reduction in TSV number in the cell-culture media as determined by real time RT-PCR indicate that TSV did not replicate in the primate cell lines used in our study. That TSV infection had occurred in SPF indicator shrimp after injection with media collected from day 7 cell culture flasks indicates that sufficient residual TSV remained in the media to infect the challenged shrimp and to cause acute disease or subacute disease as a function of relative concentration of residual TSV present.
